# A New Motor

**Published:** 1875-08

**Authors:** 


### ARTICLE VI.

### *A New "Motor."*

The alleged discovery of a new motor by a Mr. Keely, of Philadelphia, has been exciting the curiosity of scientific and unscientific men for some time past. There are those who consider it not so much a new motor as a chimera, which is an assumption formerly arrived at in regard to steam; the electric telegraph, and ocean navigation, before they were established by actual experiment. There are others who insist that it is the greatest discoveery of the age, and is going to revolutionize the forces now employed in industrial progress. It is unnecessary to say that just as sanguine expectations have been expressed before of the results of inventions which have utterly failed to realize the predictions of their patrons.

Mr. Keely, we are told, however, is about to demonstrate the entire practicability of his discovery in its application to a railroad train between Camden and Jersey City. Any expression of judgment, either for or against, until we have this practical demonstration must be premature. The public can afford to wait till Mr. Keely submits his case to actual experiment.

It is difficult to convey an intelligent conception of what the alleged new discovery is. The motive principle is represented by the New York Daily Bulletin "to be the expansive power of carbonic acid. This important compound under a pressure of thirty-six atmospheres at the temperature of thirty-two becomes liquid; and when the pressure which retains it in the liquid state is renewed the rapidity of the evaporation, and the enormous expansion of the vapor, are such as to produce a degree of cold under which acid solidifies, forming a white concrete substance possessed of very extraordinary properties. Prof. Faraday was the first who liquified carbonic acid, but it was first treated as solid by W. Thilenrier. At common temperature and pressure water absorbs its own volume of carbonic acid; under a pressure of two atmospheres it dissolves twice its volume, and so on." An intelligent correspondent of the Savannah News, who claims to have made numerous and expensive experiments with it, has no doubt of its immense power as a mechanical agent, in proof of which he adduces the following progressive augmentation of its expansive properties by increased temperatures:

At 5 degrees Fahrenheit the pressure in pounds per square inch is 372; at 10 degrees Fahrenheit the pressure in pounds per square inch is 403; at 20 degrees Fahrenheit the pressure in pounds per square inch is 460; at 30 degrees Fahrenheit the pressure in pounds per square inch is 560; at 40 degrees Fahrenheit the pressure in pounds per square inch is 697; at 45 degrees Fahrenheit the pressure in pounds per square inch is 1,080.

Heretofore the great scientific difficulty has been to know how to utilize this wonderfully expansive power—to put the harness on it, sò to speak, and to work it, as Professor Morse has harnessed electricity, and compelled it to carry messages. Now, Mr. Keely claims, this difficulty has at length been conquered, and people who profess to have patiently examined his plans, and listened to the description of his generator, multiplier, receiver, pipes, etc., are confident he will succeed. The advantages claimed for it over steam and the steam engine are many, not the least of which are economy, safety and easy control. If applied to navigation, the propelling power, it is said, would not cost more than five dollars to run a steamer from Savannah to New York; and as the necessary machinery will not take up one-fourth part of the weight and room of a boiler, engine and fuel, of equal power, another advantage would be additional carrying capacity and space for freight.

If Mr. Keely should be able to demonstrate all that he promises, the mills and manufactories and the commercial and social progress of the world would receive a new impetus. Even steam and electricity have failed to satisfy the impatient temper and the industrial demands of men, and if there are any other forces in nature which can be availed of, they will be largely pressed into service. But what is to be the lessened value of all our coal fields, if this new motor has all the virtues claimed for it?—*Baltimore Sun.*

